# Spatial distribution of breast cancer mortality: Socioeconomic disparities and access to treatment in the state of Parana, Brazil

**DOI:** 10.1371/journal.pone.0205253

**Published:** 2018-10-31

**Authors:** Sheila Cristina Rocha-Brischiliari, Luciano Andrade, Oscar Kenji Nihei, Adriano Brischiliari, Michele dos Santos Hortelan, Maria Dalva de Barros Carvalho, Sandra Marisa Pelloso

**Affiliations:** 1 Health Science Center, State University of Maringa, Maringa, Parana, Brazil; 2 Department of Medicine, State University of Maringa, Maringa, Parana, Brazil; 3 Department of Nursing, State University of the West of Parana, Foz do Iguaçu, Parana, Brazil; 4 Department of Nursing, State University of Maringa, Maringa, Parana, Brazil; 5 Public Health in the Region of Frontier, State University of the West of Parana, Foz do Iguaçu, Parana, Brazil; 6 Department of Post Graduate in Health Science, State University of Maringa, Maringa, Parana, Brazil; 7 Department of Post Graduate in Health Science and Nursing, State University of Maringa, Maringa, Parana, Brazil; University of Kentucky, UNITED STATES

## Abstract

**Introduction:**

Breast cancer remains an important public health problem that is responsible for high morbidity and mortality rates, especially in developing countries.

**Objective:**

To analyze the socioeconomic and access disparities related to breast cancer mortality in 399 cities in the state of Parana, Brazil.

**Methods:**

Ecological, descriptive and analytical cross-sectional study based on secondary data from the Mortality Information System from 2009 to 2012 in the state of Parana. Breast cancer mortality rate was calculated considering the mortality cases and women population of each municipality, both based on women older than 20 years old. Moran global and local analyses were used to verify the presence of spatial autocorrelation and spatial regression modeling (Spatial Lag—SAR) with the purpose of analyzing the association between socioeconomic indicators, access and mortality rates for breast cancer.

**Results:**

Significant positive spatial autocorrelation was found for breast cancer mortality rates (I = 0.5432, p = 0.001). In the spatial regression analysis, the model explained 61% of the variance of the mortality rates for breast cancer. The mortality rate for breast cancer was negatively associated with the illiteracy rate (Coefficient = -0.0279) and positively associated with the access index (Coefficient = 12.9525).

**Conclusion:**

The lower illiteracy rate has not been sufficient to reduce the specific mortality rate by breast cancer, and the higher the score of accessibility to cancer services, the higher the specific mortality due to breast cancer. The results show that in the state of Parana, the problem is not related to a lack of education of the patients or the distance walked, but rather with the organization of services. These conclusions have important political implications on the organization and quality of the services provided for the diagnosis and treatment of breast cancer in the state of Parana.

## Introduction

Breast cancer (CA) remains the leading cause of death among women worldwide [1–3]. In Latin America, the survival rate of breast CA is approximately 70% [4]. Developing countries such as Brazil are subjected to serious problems of access to health services, diagnosis and modern treatments. Only 20% to 50% of patients in low- and middle-income countries are diagnosed in stages I and II, while in high-income countries, 70% of cases are diagnosed in early stages [5–6]. In Brazil, in 2015, 15,403 deaths occurred due to breast CA, and 59,700 new cases of breast CA is estimated to occur in the period of 2018–2019, achieving a estimated risk rate of 56 cases per 100,000 women [7–8].

While racial disparities have been investigated extensively, little is known about the variation in breast CA mortality in different socioeconomic contexts and geographic regions, especially in small areas, such as municipalities [9]. Two factors contribute to health disparities: access to health services and inequalities in the care received [10]. For each country’s health system, specific access barriers need to be identified in different regions and subregions to eliminate these disparities and optimize timely care [6].

Brazil is the 5^th^ largest country in the world and presents accentuated regional diversity which affects the accessibility to proper diagnosis and treatment of patients with breast CA. Geographical educational diversity may affects population´s breast CA awareness, delaying the diagnosis. In addition, in Brazil, regional socioeconomic diversity is responsible for the geographical irregular distribution of mammography machines and regional patients’ restrictions to access radiotherapy and modern CA therapies. Moreover, in Brazil, higher percentage of advanced breast CA was detected in the public health system´s patients in comparison to those of the private health system, further indicating the socioeconomic diversity impact on breast CA diagnosis and treatment [11].

Although demographic data on factors that contribute to breast CA mortality are available, local patterns and information are often overlooked [12]. Studies on access barriers are scarce though essential in developing countries, where delays in CA treatment and other life-threatening diseases are very common [6].

Information on geographical patterns and temporal disease trends for different regional scales are important for the design, implementation and evaluation of CA control programs [13]. Can help to identify new hypotheses of exposure that deserve future epidemiological investigations and the with the development of better interventions and health policies [14]

In addition, studies on diseases focusing on their spatial location allow us to identify the causal relationships related to the environment, the use of health services or the behavioral analysis of the users [15]. In this sense, research on access barriers and quality of care for the diagnosis and treatment of breast CA is almost non-existent in developing countries [6].

In Brazil, the Unified Health System (SUS) provides specialized cancer care with a reference network for diagnosis and treatment distributed in different locations in the national territory [16]. A spatial analysis of this network can provide a precise geographic representation of the variation that exists for the treatment of breast CA in the state of Parana, Brazil and may point to a more effective planning strategy for the accessibility to diagnosis and treatment of breast CA. These methods and findings could also apply throughout the country.

Graphically representing the spatial distribution of breast CA mortality and investigating the influence of external factors such as access to treatment and the socio-demographic variables of the municipalities can unveil relationships not yet explored for breast CA mortality. These insights may also evoke strategies for the effective understanding of actions and interventions that will minimize the incidence of mortality from breast CA. Thus, the objective of this research was to spatially analyze breast CA mortality rates and their relationship with socioeconomic disparities and access to specialized care in 399 cities in the state of Parana, Brazil.

## Methods

### Study design and population

A retrospective and cross-sectional ecological study was performed using spatial analysis techniques based on mortality rate breast CA data from 2009 to 2012 in the state of Parana, Brazil was performed.

### Characteristics of the state of Parana

The state of Parana has a territory of 199,880 km^2^, divided into 399 municipalities. The estimated population is 10,444,526 inhabitants, most of whom (85.3%) live in urban areas, making Parana the 6^th^ most densely populated state in Brazil (5.5% of the total population). Approximately 2,5% of the municipalities have a population above 150,000 inhabitants, while other 76,7% have 20,000 inhabitants or fewer [17,18]. In 2010, Parana’s Human Development Index (HDI) was 0.749 (5^th^ in the country) [19].

The map with the cartographic base containing the political-administrative division of the state of Parana was freely obtained in shapefile format (SHP) through online access to the website of the Brazilian Institute of Geography and Statistics (IBGE) [20].

**Fig 1:**
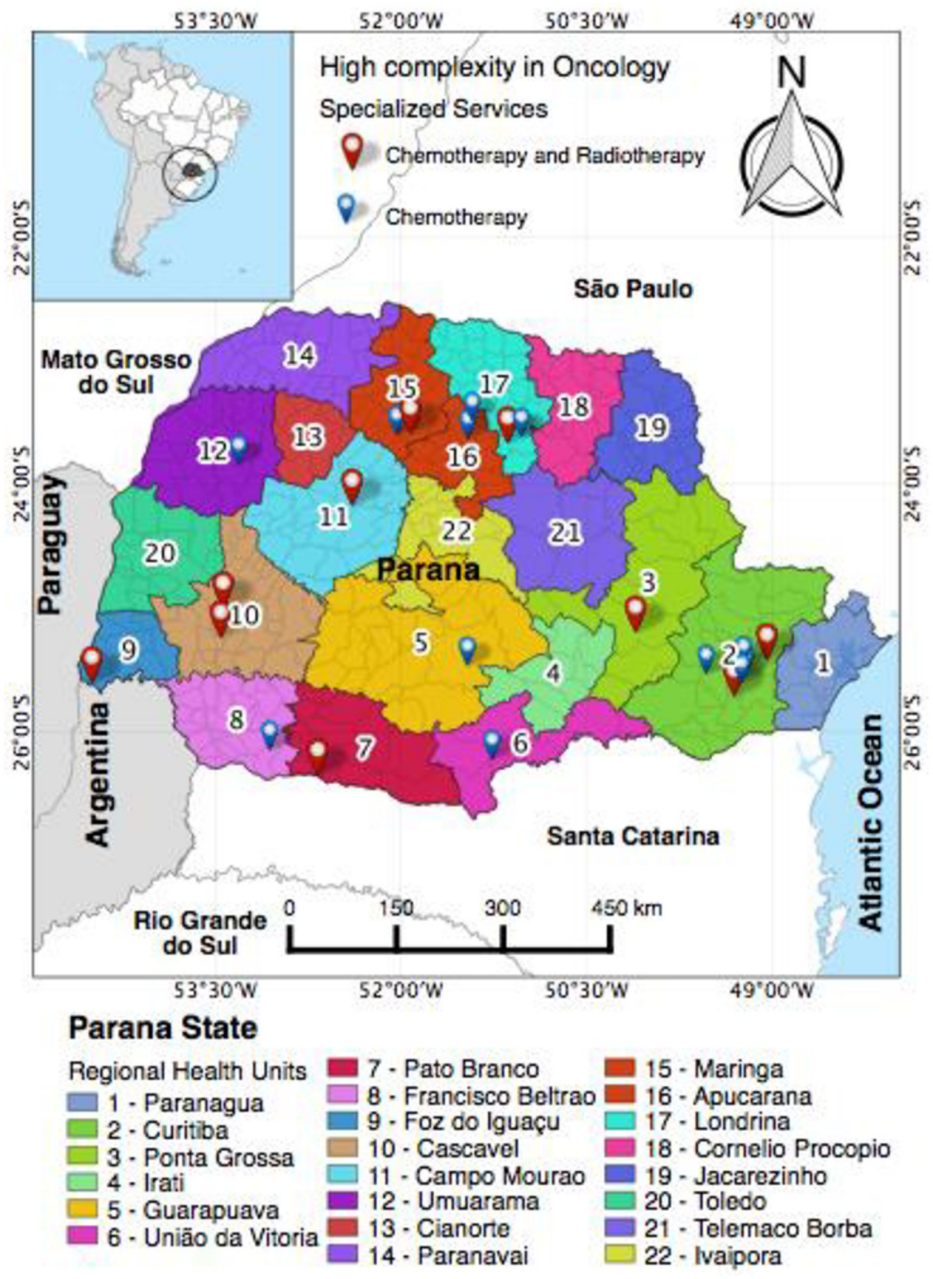
Map of the state of Parana showing the distribution of specialized services for cancer treatment in Regional Health Centers. (A) Map of the state of Parana. **Source:** Geographic Atlas of state of Parana 2008 [17]. (B) Regional Health Centers in the State of Parana and cancer care services with radiotherapy and chemotherapy. The red dots indicate radiotherapy and chemotherapy services, and the blue dots indicate services with chemotherapy. Source: Health Secretary of the state of Parana, 2012 [16].

In total, 22 Regional Health Centers (RH) are distributed across the state’s municipalities (Fig 1). The treatment of breast CA in the SUS is centralized in some reference hospitals in the state, with 10 treatment centers for chemotherapy and radiotherapy located in 2^th^, 3^th^, 7^th^, 9^th^, 10^th^, 11^th^, 15^th^ and 17^th^ RH (Map 1, points in red); and 11 treatment centers that present only chemotherapy, located in 2^th^, 5^th^, 6^th^, 8^th^, 12^th^, 15^th^, 16^th^ and 17^th^ RH [21] Fig 1. Thus, out of the 22 Regional Health Centers, only 13 provide specialized services for the treatment of CA [22] Fig 1.

### Data sources

#### Population data

Demographic data were obtained from the (IBGE) [17]. The target population was calculated by considering the total number of women older than 20 years, in each city during the analyzed time period and dividing the result by the number of years defined for the study, yielding the average number of women per city for the years 2009 to 2012.

#### Female breast cancer mortality data and specific mortality rate

Mortality data by breast CA were obtained from the Mortality Information System of the Ministry of Health (MIS/MH) [23]. The study included cases of breast CA mortality with the classification C50 (mortality by breast CA) according to the 10^th^ revision of the International Classification of Diseases (ICD-10) [24] in women older than 20 years from 2009 to 2012.

In the present study, the specific mortality rate (SMR) by breast CA in the municipalities of the state of Parana was obtained by dividing the average number of breast CA deaths, during the period of 2009 to 2012, in women older than 20 years by the average population of women in the municipality during the same period, and multiplying the result by 100,000.

An empirical bayesian spatial estimator was used to minimize random variations in breast CA mortality rates by city, mainly in municipalities with small populations, thereby eliminating the discrepancies resulting from population variation. This estimator calculates a weighted average of the gross rate of the locality and the overall rate of the region (ratio between the total number of cases and the total population) [25].

#### Socioeconomic and demographic indicators

Socioeconomic data were obtained by consulting the online public database of the IBGE [17] and the Parana Institute of Economic and Social Development (IPARDES) [18]. To establish a death profile related to breast CA, socioeconomic and demographic factors were assessed according to the patients’ residing municipality. Four socioeconomic and demographic indicators were obtained and analyzed for each city: Illiteracy (percentage of illiterate people aged 15 years or older), Income (per capita income) [17], Urbanization Degree (UD) [18], and Human Development Index (HDI) of the municipality (represents the city’s performance in relation to public management and actions, considering four parameters: employment, income, health, education) [19].

#### Distance to access breast cancer treatment

Regarding the distances traveled by the patients to access the breast CA treatment, (chemotherapy and/or radiotherapy) and mammography for diagnostic purposes, it were considered the distance from their municipality of residence to the nearest oncological referral service.

The identification of the references centers for the treatment of breast CA in the state were obtained from the regionalization directorial plan of the state of Parana. This plan establishes the final strategy for the process of identification and recognition of the health care network in its various lines of care, organized in terms of the access flow to the diagnosis and treatment of CA [21] in the territories of the State´s Regional Health Centers.

The origin-destination, the distance between home and service, defines a connection, and the number of people moving to the service determines the flow of assistance. The Network Analyst Extension of ArcGis 10.2 was used to define the distances traveled [26] using the measurement of the centroid of each city to the referral services for oncology.

#### Access to diagnosis and treatment of breast cancer: 2SFCA method

The availability and proximity of breast cancer diagnosis and treatment centers were estimated by means of accessibility index that was calculated by the two-step floating catchment area method (2SFCA) using ArcGis 10.2, which evaluates health access through a measure of spatial accessibility (availability and proximity) in a single index. This index allows comparisons in different locations with different structures between the supply and demand of health services [27]. This method uses floating coverage areas that cross static boundaries (399 municipalities of the Parana state) and overlap, allowing the modeling and measurement of health access by proximity and availability in the geographical area [27].

The 2SFCA has two basic steps: In the first stage, the areas of concentration in populations were identified within the vicinity of a particular health care provider, establishing the reach, in terms of available provider care, within a given driving time or radius of distance (buffer). For this study, these data were represented by the coverage of oncological care according to the health regionalization master plan, which includes the coverage of care in the 22 Regional Health Centers of the state. Thus, the proportion between the provider’s attendance capacity for specific communities (provider-population ratio) was calculated based on the geographic distance between the address of the CA reference center and the municipality (centroid) of the patient.

In the second stage, a buffer (radius) of 50 km was created from the centroid of each municipality, thus determining which reference services are located within the catchment of each area of population concentration. A previously calculated proportion of the provider by population residing within that radius was also obtained. This information was used to calculate the accessibility index, a summary of all the provider-population proportions [28].

### Statistical analyses

#### Spatial analysis

Exploratory spatial data analysis (ESDA) was performed using the free software GeoDa, version 0.9.5-i (Laboratory of Spatial Analysis of the University of Illinois, Urbana-Champaign, IL, USA), and the measures of global and local spatial autocorrelation (LISA—local indicators of spatial association) were determined [29]. The spatial data grouped by geographic areas (polygons) to evaluate the presence of spatial autocorrelation [30–31]. To evaluate the existence of spatial autocorrelation, a weight of the spatial matrix W was defined. This matrix allows the measurement of the non-random association between the value of a variable observed in a given geographic unit and the value of the variables observed in neighboring units. The matrix used for this study was the Queen type, which assigns a value of 1 to the neighbors in any spatial location within the analyzed area [32].

Spatial autocorrelation was obtained through univariate analysis of the (I) Moran Global Index [32–33]. This index measures both the spatial autocorrelation and the weighted neighborhood matrix, indicating that breast CA mortality rates in a given region may be similar to those in neighboring regions. Moran’s I values range from -1 to +1. Values greater or less than the Moran’s I expected value [E(I) = -1 / (n—1)] indicate a positive or negative autocorrelation, respectively. When the Moran’s I value approaches 0 (zero) indicates that the evaluated variable tend to present a random spatial distribution or spatial independence in the analyzed the areas (polygons) [32–33].

Moran’s I values between 0 and 1 indicates positive spatial association. This indicates that regions with high rates are surrounded by regions that also have high rates (High/High) and regions with low rates are surrounded by neighboring regions, which also have low (Low/Low) rates. Negative Moran’s I values (0 to -1) indicate negative spatial association. In this case, regions with high rates are surrounded by regions with low rates and *vice versa* [30, 33–34].

One limitation of Moran’s I analysis is that it can hide local patterns of spatial association [29]. To identify patterns of spatial association that were significant and specific to each area analyzed, local spatial association indicators (LISA) were used. LISA analysis allowed the identification of the existence of spatial clusters or regions with high or low values for the analyzed variables. This methodology allows the identification of regions that contribute to spatial autocorrelation [28].

Choropleth maps were generated through the open access software QGIS version 2.16 [34] for the visualization of the clusters of municipalities according to the breast CA mortality rate. These values were divided by class intervals (quartiles). The global and local spatial correlation coefficients were considered significant when p<0.05. These coefficients were analyzed by permutation levels of areas; in other words, they were confirmed by redistribution of simulated values (permutation tests) [35].

#### Multivariate spatial regression analysis

To identify which socioeconomic and geographic variables had the greatest impact on the spatial distribution of SMR by breast CA in the state of Parana, a multivariate spatial regression analysis was performed [36–37]. Initially, using the ordinary least squares (OLS) regression analysis, all independent variables (Illiteracy, Income, Urbanization Degree (UD), Human Development Index (HDI) and Accessibility Index) were included. In addition, to improve the model’s performance, we iteratively conducted a sensitivity analysis and chose the model with the best Akaike information criterion (AIC)/Bayesian information criterion values and the lowest multicollinearity. Based on this sensitivity analysis, the UD and HDI variables were excluded from the model. Since the OLS model presented a low R-square (0.1177) and presented a significant Robust Lagrange Multiplier-Lag (p<0.001) and a non-significant Robust Lagrange Multiplier-Error (p = 0.5023), a Spatial Autoregressive Lag (SAR) Model was conducted to estimate the regression coefficients of the remaining independent variables (Illiteracy, Income and Accessibility Index to breast CA diagnosis) in relation to the dependent variable (SMR for breast CA) as a weighted average with a spatially deducible variable (lag) using a Spatial weight matrix. In addition, in comparison to OLS model (AIC = 1455.67), the Spatial Lag model presented a better AIC (AIC = 1202.78).

### Ethical considerations

The study was approved by the Research Ethics Committee Involving Human Beings of the State University of West of Parana (UNIOESTE), Brazil. (Process number 1,310,870/2015).

## Results

From 2009 to 2012, there were 2215 deaths due to breast CA, an average of 553.80 +/- 17.30 deaths/year in the state of Parana. Of this total, 1278 deaths (57.7%), the majority of cases, involved women with over 8 years of schooling.

**Fig 2:**
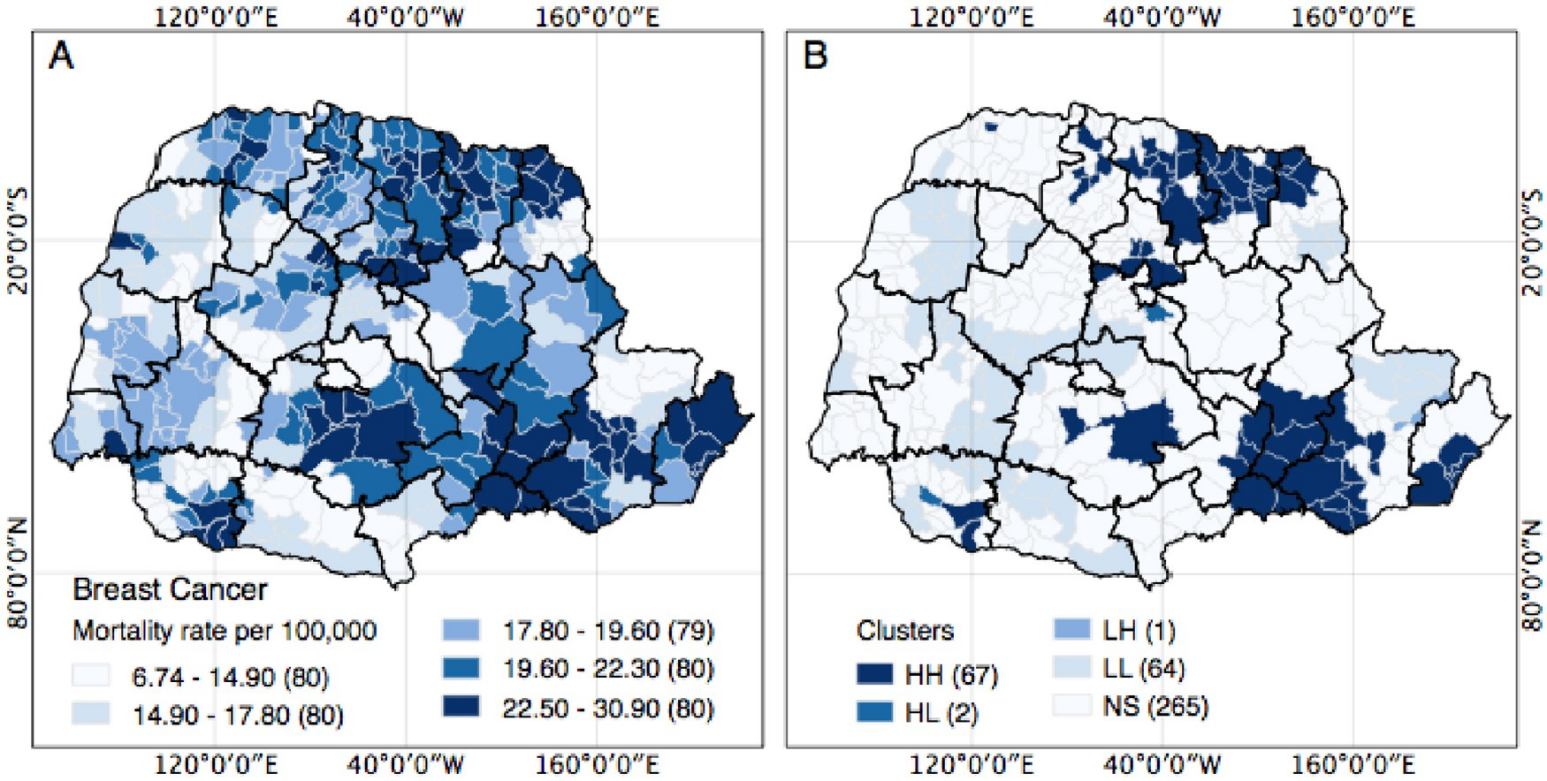
Exploratory spatial analysis of the specific mortality rate (SMR) of breast CA cases in the 399 municipalities of the state of Parana, Brazil, 2009–2012. (A) Spatial distribution of the municipalities according to their SMR by breast CA categorized according to the range of quartiles of breast CA mortality; the number of municipalities in each interval is in parentheses; (B) Univariate LISA analysis according to SMR by breast CA and identification of clusters of municipalities of the following types: high-high (HH), low-low (LL), low-high (LH) and high-low (HL).

Regarding the spatial patterns of distribution of breast CA deaths in the 399 municipalities in the state of Parana, on average, there was a SMR of 18.70 deaths/100,000 inhabitants, in patients over 20 years of age. In the analyzed period, 80 (20.05%) municipalities presented SMR by breast CA between 22.50 and 30.90 deaths per 100,000 inhabitants Fig 2A. These municipalities are located mainly in the Southeast, North, Northeast and Middle-South regions of Parana. It was also found that 80 (20.01%) municipalities had a breast CA SMR of 19.60 to 22.30 deaths per 100,000 inhabitants, 79 (19.7%) municipalities had a breast CA SMR of 17.80 to 19.60 deaths per 100,000 inhabitants, 80 (20.01%) municipalities had a SMR of 14.90 to 17.80 deaths per 100,000 inhabitants, and 80 (20.01%) municipalities had SMR of 6.74 to 14.90 deaths per 100,000 inhabitants Fig 2A.

The univariate Moran Global analysis of breast CA SMR indicated the presence of significant positive spatial autocorrelation (I = 0.5433, p = 0.001), demonstrating that municipalities with high rates of breast CA SMR are not randomly distributed in space and that these municipalities with high breast CA SMR are surrounded by other municipalities also with high breast CA SMR.

LISA analysis allowed the detection of clustering based on similarities between the municipalities according to SMR by breast CA Fig 2B. Through this analysis, municipalities were grouped according to the following categories: 1) High-High (HH), cities with high rates of death by breast CA with neighboring areas also showing high mortality rates by breast CA; 2) Low-Low (LL), cities with low rates of breast CA mortality with neighboring areas also showing low mortality rates by breast CA; 3) Low-High (LH), cities with low rates of breast CA with neighboring areas showing high mortality rates by breast CA; and 4) High-Low (HL), cities with high mortality rates by breast CA with neighboring areas showing low mortality rates by breast CA Fig 2B.

Sixty-seven municipalities showed HH clusters (Fig 2B), including the following Regional Health (RH): 1^st^ RH (four cities), 2^nd^ RH (ten cities), 3^rd^ RH (four cities), 4^th^ RH (three cities); 5^th^ RH (two cities), 6^th^ RH (two cities); 8^th^ RH (two cities), 14^th^ RH (one city), 15^th^ RH (five cities); 16^th^ RH (four cities), 17^th^ RH (ten cities); 18th RH (fifteen cities); 19^th^ RH (four cities) and 22^nd^ RH (one city).

In addition, 64 municipalities were categorized as low-low (LL) clusters Fig 2B: RH 2^nd^, 5^th^, 7^th^, 8^th^, 10^th^, 11^st^, 12^nd^, 13^rd^, 14^th^, 19^th^, 20^th^ and 22^nd^ RH. One municipality, in the 2^nd^ RH, was categorized as low-high (LH) (Fig 2B), and two municipalities, in the 8^th^ RH and 22^nd^ RH, were identified as High-Low (HL) Fig 2B.

Table 1 presents the results of the spatial regression analysis to identify the socioeconomic and accessibility variables related to breast CA SMR. Through this analysis, it was found that out of the ther predictors analyzed, two were significantly associated with breast CA SMR (p <0.05). In addition, the applied model explained 58% of the spatial dependence variance for mortality by breast CA. The positive correlation for the ‘Accessibility score’ (Coefficient = 12.9527; p = 0.014) indicates that municipalities that present high SMR by breast CA present, on average, neighbors with greater geographical accessibility for radiotherapy or chemotherapy This result suggests that the mortality by breast CA is not associated with the lack of geographical access to specialized services in the local or regional level. Thus, in these settings, other factors such as care flow, population density and/or organization of health services may be more prevalent for mortality than the supply of care Table 1. There was a negative correlation for the variable ‘Illiteracy rate’ (Coefficient = -0.0279; p = 0.029) Table 1. This result indicates that local and neighbors’ low illiteracy rates is associated with high rates of mortality by breast CA suggesting that education is not a protective factor for the disease.

**Table 1:** Results of Spatial Lag regression analysis of socioeconomic variables and accessibility index in relation to breast cancer specific mortality rate, 2009–2012. Parana, Brazil.

Spatial Lag Model	Coefficients	P value
Income	-0.0011	0.887
Illiteracy rate	-0.0279	0.029^a^
Accessibility index	12.9527	0.014^a^

Spatial Lag Model’s R-squared = 0.6117

^a^ Statistically significant (p<0.05)

Stated differently, socioeconomic factors significantly influence SMR by breast CA in these cities and, therefore, may be related to the grouping pattern observed in the study.

**Fig 3:**
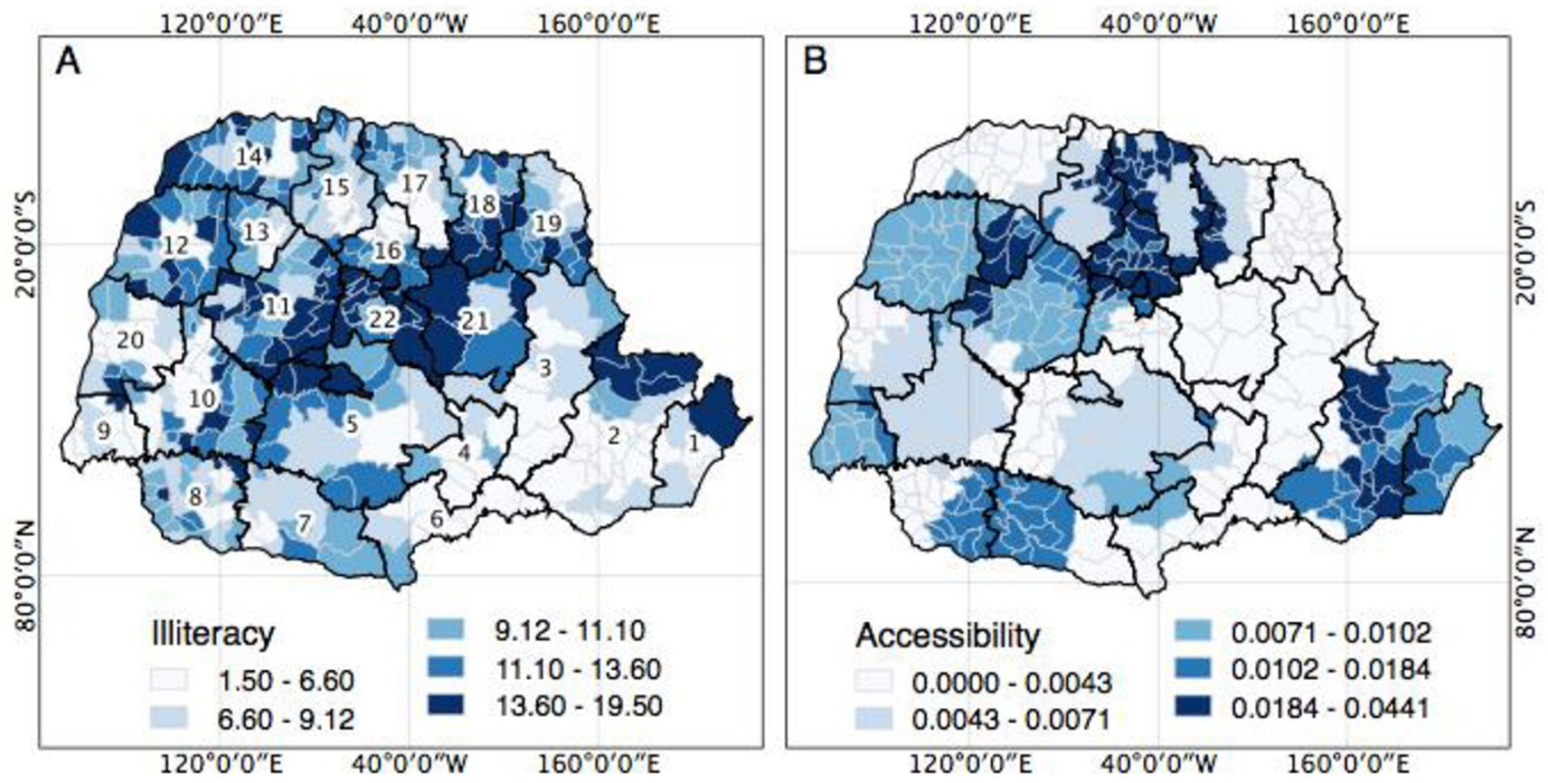
Spatial distribution of socioeconomic variables and related to access to oncology services in the 399 municipalities of the state of Parana, Brazil, 2009 to 2012. (A) Spatial distribution of municipalities according to illiteracy rate, categorized according to the range of quartiles; (B) Spatial distribution of municipalities according to their score of accessibility to chemotherapy and radiotherapy services categorized according to ranges of quartiles.

Fig 3 shows the spatial distribution of the illiteracy rate Fig 3A. and the accessibility score for health services (radiotherapy and chemotherapy) Fig 3B. in the 399 municipalities of the state of Parana. From 2009 to 2012, municipalities with high illiteracy rates were mainly located in geographical regions with municipalities with low SMR by breast CA Fig 3A. As presented in Table 1, illiteracy rate showed an inverse correlation with SMR by breast CA.

In addition, municipalities with high accessibility scores were located geographically close to the regions of the state where municipalities with high SMR by breast CA are located Fig 3B. As presented in Table 1, a positive correlation was found between the accessibility score and SMR by breast CA.

## Discussion

To our knowledge, in developing countries and more specifically in Latin America, this is the first study to evaluate the relationship between deaths due to breast CA and socioeconomic and demographic conditions through methodologies related to ecological studies.

Recorded data on CA are valuable because they provide the opportunity to identify geographic information using mapping technologies to identify sites where further investigation and intervention are needed to reduce the incidence and mortality by breast CA [38]. Spatial analysis can reveal different risk factors and contribute significantly to establishing policies to combat CA in different areas and population groups [39].

The present study demonstrated the presence of significant positive spatial dependence among the 399 municipalities in the state of Parana, Brazil with regard to SMR by breast CA, i.e., cities with high SMR by breast CA were surrounded by cities with high SMR by breast CA, suggesting a high-high spatial grouping pattern. Likewise, municipalities with low-low-type clusters were identified, thus indicating the presence of spatial discrepancies regarding the rates of breast CA deaths in the state.

A previous study indicated that socioeconomic status plays an important role in mortality by breast CA [40]. The present study found a negative association between the illiteracy rate of the population and SMR by breast CA. Thus, better education conditions may be related to variables that may influence the SMR by breast CA. These data reveal a different scenario from some previous studies. As an example, high poverty and low schooling have been related to poor access to health services [41]. Thus, the low socioeconomic level results in a low frequency of screening for this group of women, making it a target for intervention and health policies aimed at reducing socioeconomic inequalities [42] because it contributes to raising morbidity and mortality by this cause.

A recent study that investigated national socioeconomic development as verified by the Global Human Development Index (HDI) showed that low socioeconomic status had a negative effect on breast CA, especially in developing countries with a lower HDI [43]. In California, it was found that low socioeconomic status was a significant risk factor for mortality by breast CA across the state, while other related variables vary by location [44].

Thus, the results of this study differ from previous findings. Considering that Brazil presents different regional scenarios in the result obtained for the state of Parana, which is located in the southern region of the country, our results may reflect a scenario where illiteracy rates are on average reduced and may indicate the influence of other variables that are indirectly associated with the breast CA.

In the present study, a high score of accessibility to oncology services (radiotherapy and chemotherapy) was positively correlated with SMR by breast CA. The fact that the present study indicates that the accessibility score is positively associated with SMR by breast CA indicates that the municipalities with higher SMR by breast CA are close to the services of specialized treatment in oncology and in places with greater population density.

However, this result differs from the findings of a previous study that investigated the relationship between geographical disparities and the final stage of diagnosis for breast cancer incidence. This previous study showed that diagnosis was more common in areas with a predominantly black population, lower illiteracy, and low availability of screening [38].

It is notable that women living in areas with higher population concentrations have greater difficulty accessing breast cancer treatment, which and may indicate a direct relation with mortality by breast CA in these locations. Similar data have been reported in a study of breast CA screening among native Indians and Alaskan natives [45].

Additionally, with regard to access, a study conducted in South and Central America has shown, among other factors, that inequalities in access and coverage, low resources and inadequate infrastructure are determinants for increasing mortality of breast CA [46].

However, despite the existing studies, it must be noted that it has not been established to what extent the disparities in health care services affect CA mortality in women [43]. Although breast-related diseases, especially breast cancer, are more common in countries with limited resources, administrative and clinical capacity must manage all necessary care in the referenced areas [47].

In those places identified as having high priority, access to specialized services should be increased to reduce disparities [13] regarding mortality and to equalize conditions of access to equal the services of other centers. Thus, once a problem has been identified, specific resources can be targeted to minimize risk factors that contribute to mortality in a specific way within the community, thereby increasing the likelihood of a beneficial outcome for the population as a whole [12].

Although breast CA control policies prioritize access, they fail to identify specific strategies for adherence to treatment [48]. Providing specialized treatment for breast CA is one of the priorities of health managers in Brazil and throughout the world, but studies that analyze the social determinants of health [45], such as the impact of socioeconomic status and access to cancer services, are still scarce [49].

To reduce breast CA mortality in all segments of the population, it is necessary to define which populations have the greatest need for interventions and to characterize the disparities of the underlying risk factors contributing to increased mortality [12]. Thus, mapping the spatial and temporal distributions of the disease is directly related to human interventions [14] and contributes to measures of control of breast CA.

Thus, increasing availability and improving access to adjuvant breast CA therapy for women who are at higher risk of not receiving such treatment because of ethnicity, geography or socioeconomic status should be prioritized, which may help minimize the inequalities of breast CA [50].

As in the present study, it was found that the accessibility score to specialized service showed a positive correlation with SMR by breast CA in the municipalities of the state of Parana, this result raises questions about the quality of services provided, availability of access, and number of professionals and training. These factors may constitute an important focus of future research on the impact of the services provided in the public health sector in SMR by breast CA. As study limitations, the CA mortality rate was based on secondary database, thus we cannot discard the possibility of underreporting data, in this case, meaning that the real CA SMR in the Parana state may be even higher compared to that found in the present study. Another limitation is related to cross-sectional studies which do not allows the control of exposition to independent variables, in this study we cannot infer any direct causality related to the evaluated independent variables, but, as our model indicates, significant associations were demonstrated.

It is concluded from the SMR of breast cancer in Parana with regard to illiteracy rate that breast CA is higher among women with higher education.

Access to referral centers for cancer therapy was positively associated with SMR by breast CA; that is, the higher the accessibility score to oncology services, the greater the specific mortality due to breast cancer in the state. This conclusion has important political implications not only in the distribution of breast CA treatment access within the care networks across Brazil but also with regard to the quality of the services offered and the training of oncology care professionals in the state. It is necessary to determine with more scientific rigor if the oncology reference centers are able to supply the needed care and, moreover, if there is a quality standard for these services in the country in relation to infrastructure demands. In addition, based on the results obtained in the present study, the need to investigate other variables that may also be related to high SMR by breast CA in the state of Parana is evident, for example, behavioral factors related to the demand for the health service and adherence to treatment by the female population of the state.

These factors are crucial for the early diagnosis of breast CA and adequate treatment in scenarios where access to cancer care services is supported, as is the case for the municipalities of Parana with high SMR by breast CA and a high population density.

## Supporting information

S1 File Database available in public repository Figshare by link: https://figshare.com/account/articles/6453278. (CSV)
